# Relationship between Humoral Immune Responses against HPV16, HPV18, HPV31 and HPV45 in 12-15 Year Old Girls Receiving Cervarix^®^ or Gardasil^®^ Vaccine

**DOI:** 10.1371/journal.pone.0140926

**Published:** 2015-10-23

**Authors:** Anna Godi, Sara L. Bissett, Elizabeth Miller, Simon Beddows

**Affiliations:** 1 Virus Reference Department, Public Health England, London, United Kingdom; 2 National Vaccine Evaluation Consortium, Public Health England, London, United Kingdom; National Institute of Health - National Cancer Institute, UNITED STATES

## Abstract

**Background:**

Human papillomavirus (HPV) vaccines confer protection against the oncogenic genotypes HPV16 and HPV18 through the generation of type-specific neutralizing antibodies raised against virus-like particles (VLP) representing these genotypes. The vaccines also confer a degree of cross-protection against HPV31 and HPV45, which are genetically-related to the vaccine types HPV16 and HPV18, respectively, although the mechanism is less certain. There are a number of humoral immune measures that have been examined in relation to the HPV vaccines, including VLP binding, pseudovirus neutralization and the enumeration of memory B cells. While the specificity of responses generated against the vaccine genotypes are fairly well studied, the relationship between these measures in relation to non-vaccine genotypes is less certain.

**Methods:**

We carried out a comparative study of these immune measures against vaccine and non-vaccine genotypes using samples collected from 12–15 year old girls following immunization with three doses of either Cervarix^®^ or Gardasil^®^ HPV vaccine.

**Results:**

The relationship between neutralizing and binding antibody titers and HPV-specific memory B cell levels for the vaccine genotypes, HPV16 and HPV18, were very good. The proportion of responders approached 100% for both vaccines while the magnitude of these responses induced by Cervarix^®^ were generally higher than those following Gardasil^®^ immunization. A similar pattern was found for the non-vaccine genotype HPV31, albeit at a lower magnitude compared to its genetically-related vaccine genotype, HPV16. However, both the enumeration of memory B cells and VLP binding responses against HPV45 were poorly related to its neutralizing antibody responses. Purified IgG derived from memory B cells demonstrated specificities similar to those found in the serum, including the capacity to neutralize HPV pseudoviruses.

**Conclusions:**

These data suggest that pseudovirus neutralization should be used as the preferred humoral immune measure for studying HPV vaccine responses, particularly for non-vaccine genotypes.

## Introduction

The human papillomavirus (HPV) vaccines (Cervarix^®^ and Gardasil^®^) contain virus-like particles (VLP) comprising the major capsid protein (L1) of HPV16 and HPV18 and are highly efficacious at preventing cervical cancer precursors associated with these two high risk genotypes in clinical trials [1–3]. Gardasil^®^ also contains VLP representing the main gentoypes associated with the development of genital warts (HPV6 and HPV11). HPV16 and HPV18 account for *ca*. 70% of cervical cancers worldwide [4, 5] and a recent meta-analysis [6] of epidemiological data from Australia [7], the USA [8] and the UK [9–11] demonstrate reductions in the prevalence of these two genotypes following the introduction of national HPV vaccination programmes.

Neutralizing antibodies against vaccine genotypes can be detected in the serum and genital secretions of vaccinees [12–14] and passive transfer of neutralizing antibodies can protect animals against papillomavirus challenge [15–17] leading to the reasonable assumption that type-specific protection is mediated by neutralizing antibodies [2]. Some degree of cross-protection has been demonstrated against the non-vaccine genotypes HPV31 and HPV45 that are genetically-related to the vaccine genotypes HPV16 and HPV18, respectively [1, 3, 18, 19]. This is coincident with the detection of cross-neutralizing antibodies in the serum [14, 20–23] and cervicovaginal secretions [14] of vaccinees suggesting that such antibodies may be effectors or their detection may be useful as a correlate or surrogate of vaccine-induced cross-protection [24].

A limited number of serological assays are available for measuring vaccine-type (HPV16 and HPV18) antibody responses, including a VLP ELISA, a monoclonal antibody competitive VLP assay and a pseudovirus neutralization assay. Despite some discrepancies, overall inter-assay agreements appear to be good [25–27]. However, little is known about the relationship between these measures for non-vaccine types.

The detection of antigen-specific memory B cells may be indicative of a robust and long-lasting vaccine-induced immune response [28, 29], but relatively few studies have examined the proportion and specificity of memory B cells induced by the HPV vaccines. Early HPV studies estimated antigen-specific memory B cell frequencies induced by prototype VLP16 and/or VLP18 immunogens [30, 31] while more recent studies have assessed HPV16 and HPV18 specific memory B cell responses generated by the licensed vaccines Cervarix^®^ and Gardasil^®^ [12, 32]. One of these studies [12], carried out in 18–45 year old women, assessed binding and neutralizing antibody and memory B cell responses induced by both Cervarix^®^ and Gardasil^®^ against vaccine (HPV16 and HPV18) [12, 33, 34] and non-vaccine (HPV31 and HPV45) [22] genotypes. For all humoral immune measures, the magnitudes of the responses against HPV16 were generally greater than those responses against HPV18 with Cervarix^®^ eliciting responses of a greater magnitude than Gardasil^®^ [33]. For HPV31, and to a lesser extent for HPV45, Cervarix^®^ appeared to generate neutralizing antibody responses of a greater magnitude than Gardasil^®^, but this was not reflected in the antibody binding or memory B cell responses [22]. Some of these observations were likely affected by the older age of the women enrolled, a parameter known to affect HPV vaccine immunogenicity [35].

We recently carried out an immunogenicity trial of Cervarix^®^ and Gardasil^®^ in the target age group (12–15 year old girls) for national vaccination programmes and demonstrated high levels of serum cross-neutralizing antibodies, a clear difference between the responses generated by the vaccines and an ability to detect neutralizing antibodies against vaccine and non-vaccine genotypes in the genital secretions of vaccinated girls [14]. In the present study, we examine the breadth and magnitudes of the memory B cell responses generated by both HPV vaccines against vaccine (HPV16 and HPV18) and non-vaccine (HPV31 and HPV45) HPV genotypes for which vaccine-induced protection has been consistently observed and compare these to their serum neutralizing and binding antibody responses in order to understand better these vaccine-induced immune measures in the target age group.

## Materials and Methods

### Study samples

The study design of the randomized, observer-blinded immunogenicity trial of Cervarix^®^ and Gardasil^®^ vaccines in 12–15 year old girls and the primary serum neutralizing antibody response analysis have been reported previously (Research Ethics Committee reference: 09/H0720/25) [14]. The present study examined those individuals who consented to provide an additional blood sample at month 7 (M7) following three doses of either Cervarix^®^ or Gardasil^®^ vaccine for evaluation of their memory B cell responses. Peripheral blood mononuclear cells (PBMC) were isolated from a 20-30mL sample of heparinized blood, processed according to standard protocols and stored in liquid nitrogen. M7 serum samples from these individuals were also included in this present study.

### ELISA

L1 VLP representing HPV16, HPV18, HPV31 and HPV45 were expressed using the Bac-to-Bac^®^ Baculovirus System (Life Technologies), as previously described [14] wherein the L1 genes shared 100% amino acid sequence identity with the L1 genes of the pseudovirus clones used for the neutralization assay [23]. Serum (M7) binding antibody responses were determined for those individuals who consented to provide a PBMC sample for memory B cell determination using an L1 VLP ELISA, as described previously [14]. L1L2 pseudoviruses (see 2.3) were also used in a binding ELISA as described above.

### Neutralization assay

Serum (M7) neutralizing antibody data, previously generated [14] against HPV16, HPV18, HPV31, HPV45 pseudoviruses, were included in the present study.

### ELISpot Assay

Enumeration of memory B cells by ELISpot was carried out according to the original method of Crotty et al., [29] with minor modifications including the use of cryopreserved PBMC [36]. PBMC (1x10^6^ per mL) were rested in culture medium (RPMI 1640 with 10% fetal calf serum, 100 units/mL penicillin, 100 μg/mL streptomycin, 2mM L-Glutamine, 10mM Hepes and 1mM Sodium Pyruvate) at 37°C for one hour before being stimulated with 1μg/mL R848 (resimiquimod; MABTECH, Cincinnati, OH) and 10ng/mL recombinant human IL-2 (interleukin-2; MABTECH, Cincinnati, OH) for 5 days. The median initial PBMC viability of 93% (inter-quartile range, IQR 92–95%), as determined by trypan blue exclusion, was reduced to 86% (84–89%) following R848/IL-2 stimulation.

IgG ELISpot assays were performed using a commercially available kit (IgG ELISpot 150 plus kit (MABTECH) according to the manufacturer’s instructions. ELISpot plates were coated with 3 μg/mL HPV16, HPV18, HPV31 or HPV45 VLP (n = 3 each) [14], 15 μg/mL anti-human IgG (total IgG; MABTECH, Cincinnati, OH) (n = 3) or PBS (mock; n = 6) and incubated overnight at 4°C. The following day, 5x10^4^ PBMC were added to the VLP and mock wells and 4x10^2^ PBMC added to the anti-human IgG wells, then the plate was incubated at 37°C for 6 h. Cells were removed and the plate washed four times with PBS containing 0.05% Tween 20 (Sigma) and four times with PBS followed by incubation with anti-human IgG–horseradish peroxidase overnight at 4°C. The plate was washed as above, incubated for 1hr at RT with streptavidin-HRP followed by addition of TMB substrate solution for 15 min at RT. Spot-forming units (SFU) were counted using the AID ELISpot reader ELR04 (AID, Germany) and data are presented as the percentage of HPV-specific memory B cells per total IgG-secreting cells as standard [29]. The mock, no antigen control, was used to gauge the level of background staining. Overall there were a median 1.2 (IQR 0.7–2.0) apparent SFU per well in the mock wells resulting in an effective median background level compared to total IgG SFU of 0.025% (IQR 0.014–0.036%). For a well to be considered positive it had to have at least 5 SFU per well, equivalent to 100 SFU per 10^6^ PBMC or *ca*. 0.1% of IgG-bearing cells, and be at least 3 times higher than the mock SFU level for that individual. This is a similar approach to that taken by Walsh et al., [37] as part of a methodological evaluation for The NIAID HIV Vaccine Trials Network.

### Memory B cell derived IgG

Memory B cell derived IgG were purified (Protein G GraviTrap and Ab buffer kit; GE Healthcare Life Sciences, UK) from culture supernatant collected following R848/IL-2 stimulation. The eluted IgG were concentrated by centrifugation using an Amicon^®^ Ultra-4 Centrifugal Filter Unit (10 kDa cutoff; Millipore, UK) and human IgG levels estimated using an indirect ELISA as previously described [14]. The linearity of the standards was good with an average *r* ^*2*^ of 0.990 (s.d. 0.004; n = 3). The median level of recovered IgG was 51.3 (IQR 40.6–66.8) μg/mL.

### Data analysis

Fisher’s Exact test was used to test for differences in the proportion of individuals positive in a particular test. The Mann Whitney U test was used to test for differences in the magnitude of responses in a particular test and for any differences in the age range in the vaccinees. A non-parametric trend analysis was used to test for an association between measures for one target in one test against the neutralizing antibody responses by the HPV16 or HPV18 vaccine-genotype, as appropriate. All analyses were 2-tailed where appropriate and performed using Stata 13.1 (Statacorp, College Station, TX).

## Results

### Study participants

PBMC from eighty four consenting individuals were available. Four were excluded due to low viability resulting in PBMC from eighty (Cervarix^®^ n = 36; Gardasil^®^ n = 44) individuals being used. The ages of the individuals who received Cervarix^®^ (median 14, range 12–15 years) were similar to those who received Gardasil^®^ (14, 12–15 years; *p* = 0.461 Mann Whitney U test).

### Proportion of responders

**Fig 1:**
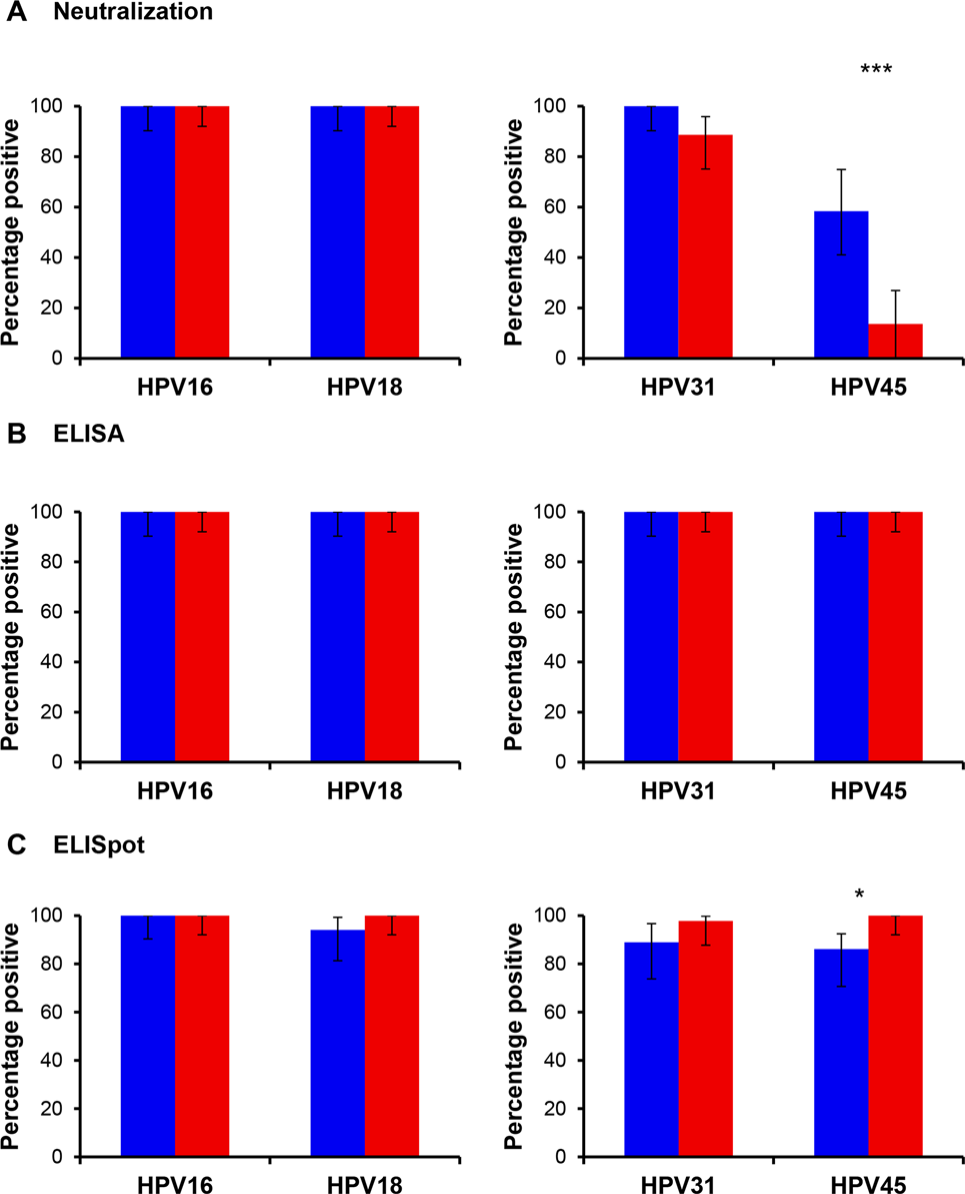
Percentage of responders to each humoral immune measure. Percentage of Cervarix^®^ (Blue) or Gardasil^®^ (Red) responders in the (A) neutralization assay, (B) binding assay or (C) B cell ELISpot assay against the indicated vaccine (HPV16, HPV18) and non-vaccine (HPV31, HPV45) genotypes. Error bars, 95% CI. * p<0.05; ** p<0.01; *** p<0.001.

Seropositivity rates for vaccine-type (HPV16 and HPV18) neutralizing and binding antibodies were 100% for individuals receiving either Cervarix^®^ or Gardasil^®^ vaccines, as expected (**Fig 1**). These were similar to the proportion of individuals with detectable memory B cell responses against HPV16 (Cervarix^®^ 100% and Gardasil^®^ 100%) and HPV18 (Cervarix^®^ 94% and Gardasil^®^ 100%). A high proportion of individuals immunized with Cervarix^®^ (100%) or Gardasil^®^ (89%) elicited a neutralizing antibody response against HPV31, with 100% of individuals generating binding antibody response against this non-vaccine genotype and almost all (Cervarix^®^, 89% and Gardasil^®^ 98%) had detectable HPV31-specific memory B cells. For HPV45, however, the relatively low and differential proportion of individuals with a measurable neutralizing antibody response following Cervarix^®^ (58%) or Gardasil^®^ (14%) vaccination was in contrast to the 100% seropositivity rates for binding antibodies and the similarly high proportion of individuals with detectable HPV45-specific memory B cells (Cervarix^®^, 86% and Gardasil^®^ 100%).

### Magnitude of humoral immune response measures

**Fig 2:**
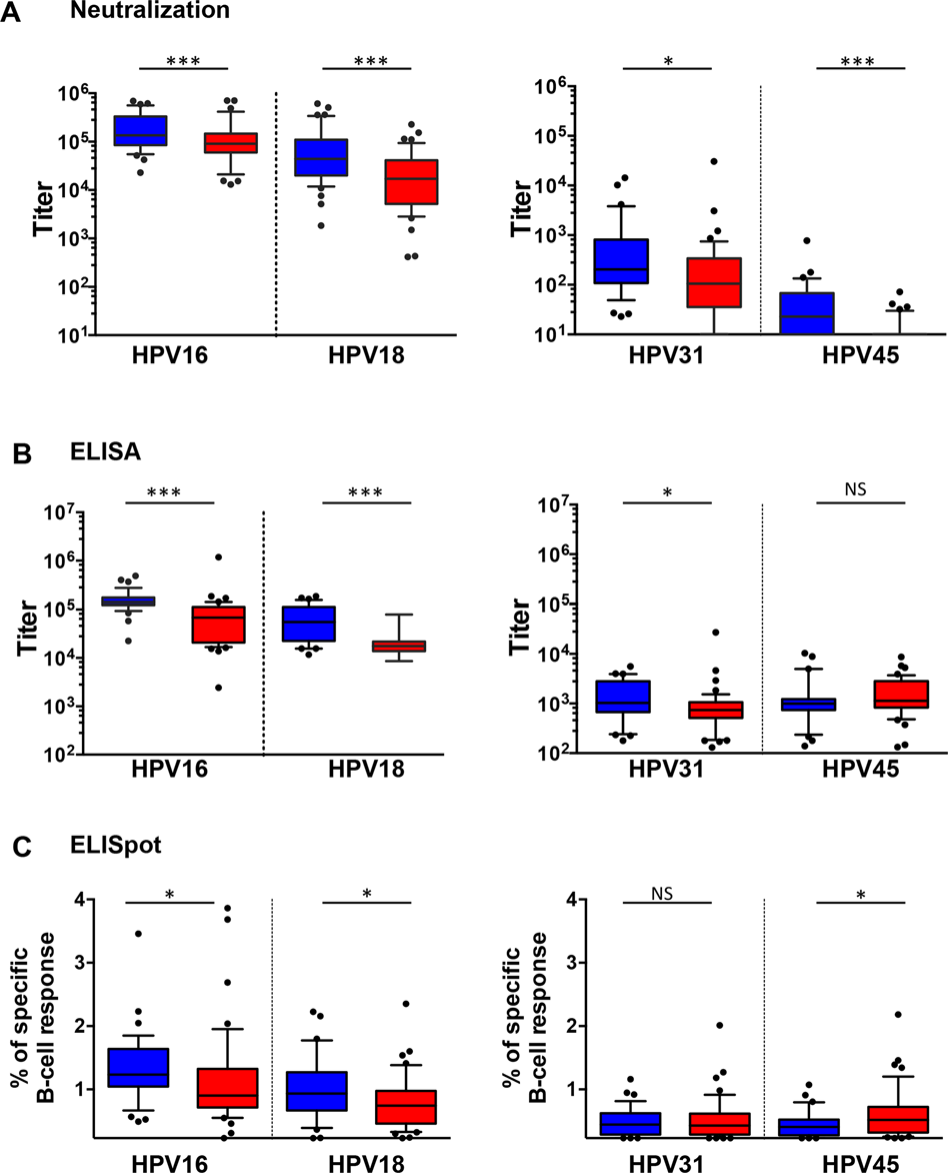
Magnitude of humoral immune responses. Box (median and IQR) and whisker (10^th^ - 90^th^ percentiles) plots of the magnitude of humoral immune responses elicited by Cervarix^®^ (Blue) and Gardasil^®^ (Red) vaccinees in the (A) neutralization assay, (B) binding assay or (C) B cell ELISpot assay against the indicated vaccine (HPV16, HPV18) and non-vaccine (HPV31, HPV45) genotypes. * p<0.05; ** p<0.01; *** p<0.001.

Serum HPV16 neutralizing antibody titers for Cervarix^®^ vaccinees (median 135,617, IQR 85,137–328,767) were higher than for those who received the Gardasil^®^ vaccine (44,048, 20,696–105,982; *p*<0.001) (**Fig 2 and S1 Table**) similar to the differential HPV18 titers for Cervarix^®^ (90,693, 63,222–142,890) and Gardasil^®^ (17,011, 5,544–40,707; *p*<0.001). Similar differences between the vaccines were seen for HPV16 (Cervarix: 140,040, 123,850–172,575 and Gardasil: 68,423, 21,355–112,534; *p*<0.001) and HPV18 (Cervarix: 55,394, 23,657–109,494 and Gardasil: 17,559, 13,792–21,814; *p*<0.001) serum binding antibody responses. The median percentage of HPV16-specific memory B cell responses for Cervarix^®^ vaccinees was 1.23% (IQR 1.07–1.61%) which was higher than the median 0.91% (0.72–1.31%; *p* = 0.034) seen in Gardasil^®^ vaccinees. HPV18-specific memory B cells were lower than for HPV16, with individuals receiving Cervarix^®^ (0.93%, 0.69–1.24%) having higher levels than Gardasil^®^ (0.75%, 0.47–0.97%; *p* = 0.021) vaccinees.

HPV31 neutralizing antibody titers were slightly higher in Cervarix^®^ (204, IQR 121–686) compared to Gardasil^®^ (112, 41–379) vaccinees (*p* = 0.019). Similarly, HPV31 binding titers were slightly higher in Cervarix^®^ (1,032, 680–2,743) compared to Gardasil^®^ (739, 533–1,060) vaccinees (*p* = 0.024). Estimates of circulating HPV31-specific memory B cells for individuals receiving Cervarix^®^ (0.44%, 0.30–0.62%) were similar to those who received Gardasil^®^ (0.43%, 0.28–0.59%; *p* = 0.954) vaccine.

HPV45 neutralizing antibody titers were higher in Cervarix^®^ (23, 10–59) compared to Gardasil^®^ (10, 10–10; *p*<0.001) vaccinees but this was not reflected in the binding titers which were similar between Cervarix^®^ (998, 754–1,230) and Gardasil^®^ (1,131, 866–2,803; *p* = 0.171) vaccinees. Estimates of circulating HPV45-specific memory B cells for individuals receiving Cervarix^®^ (0.41%, 0.28–0.51%) were slightly lower than those who received Gardasil^®^ (0.52%, 0.33–0.71%; *p* = 0.020) vaccine.

### Association with vaccine-type neutralizing antibody responses

To elucidate further any potential relationship(s) between neutralizing and binding antibody titers and the levels of memory B cells specific for vaccine and non-vaccine genotypes, we compared whether a measure increased in a stepwise manner according to the low, middle or high tertiles of the corresponding vaccine-type neutralization titers which would be suggestive of a relationship between the magnitudes of these responses. In this respect, vaccine-type specific serum binding antibody (HPV16, *p*<0.001; HPV18, *p*<0.001) and memory B cell (HPV16, *p*<0.001; HPV18, *p*<0.009) responses demonstrated close associations with their respective neutralizing antibody tertiles (**Table 1**).

**Table 1:** Comparison of non-vaccine type and vaccine-type immune measures.

			Immune responses against vaccine and non-vaccine types from indicated species groups ^a^			
Target	Immune measure	Tertile	Alpha-9 (HPV16 and HPV31)	*p* value	Alpha-7 (HPV18 and HPV45)	*p* value
**Vaccine types**	Neutralizing antibody	T1	22,960 (17,690–31,289)		11,028 (4,259–13,361)	
		T2	83,869 (71,796–104,323)		51,451 (30,028–66,105)	
		T3	329,411 (157,076–473,509)	N/A	123,701 (105,933–200,388)	N/A
	Binding antibody	T1	22,418 (18,937–59,497)		15,306 (12,257–18,448)	
		T2	112,452 (90,889–130,872)		21,387 (16,246–36,373)	
		T3	168,576 (139,593–221,501)	**<0.001**	101,046 (61,129–139,189)	**<0.001**
	Memory B cells	T1	0.80% (0.71–1.00%)		0.61% (0.48–0.97%)	
		T2	1.25% (0.99–1.64%)		0.84% (0.58–0.94%)	
		T3	1.39% (1.16–1.71%)	**<0.001**	1.07% (0.83–1.30%)	**<0.009**
**Non-vaccine types**	Neutralizing antibody	T1	93 (31–159)		10 (10–10)	
		T2	157 (80–340)		10 (10–36)	
		T3	511 (138–2,270)	**<0.001**	21 (10–49)	**<0.001**
	Binding antibody	T1	569 (258–821)		943 (749–1,234)	
		T2	974 (662–1,073)		1,057 (779–1,884)	
		T3	1,558 (798–3,352)	**<0.001**	1,188 (859–4,085)	0.062
	Memory B cells	T1	0.36% (0.26–0.47%)		0.49% (0.33–0.70%)	
		T2	0.44% (0.32–0.58%)		0.49% (0.28–0.59%)	
		T3	0.54% (0.32–0.75%)	**0.022**	0.44% (0.28–0.69%)	0.318

^*a*^ Median (inter-quartile range, IQR) for immune measures against vaccine (HPV16, HPV18) and non-vaccine (HPV31, HPV45) genotypes from the Alpha-9 (HPV16, HPV31) and Alpha-7 (HPV18, HPV45) species groups. *p* value, test for trend for each measure following separation of responses into tertiles (T1-T3) based upon vaccine-type neutralizing antibody responses. N/A, not applicable.

HPV31 neutralizing (*p*<0.001) and binding (*p*<0.001) antibody titers, and to a lesser extent memory B cell responses (p = 0.022), demonstrated close associations with the magnitude of the neutralizing antibody titers generated against the vaccine genotype, HPV16. For HPV45, however, although neutralizing antibody titers were generally related to the magnitude of the responses against the vaccine genotype HPV18 (*p*<0.001), binding antibody titers (*p* = 0.062) and memory B cell responses (*p* = 0.318) were poorly related.

### Functionality of memory B cell-derived IgG

To demonstrate that memory B cell derived IgG retained neutralizing antibody capability we purified total IgG from the culture supernatant of R848/IL-2 stimulated PBMC (n = 10) and used these in neutralization and binding assays (**Table 2**). Memory B cell derived IgG from all individuals were able to neutralize pseudoviruses representing both vaccine genotypes (HPV16 and HPV18) and this was similarly reflected in their binding capability. Memory B cell derived IgG samples were also able to neutralize non-vaccine genotypes, HPV31 and HPV45, although the inhibitory concentration required to do so was 1–2 Log_10_ higher. Nine of ten samples were able to neutralize the non-vaccine genotype HPV31 while only four of ten purified IgG samples were able to neutralize HPV45. Almost all samples were able to bind L1 VLP by ELISA in keeping with the antibody specificities seen using serum. However, when L1L2 pseudoviruses were used as the target antigen in binding assays, fewer samples were able to bind the antigen representing HPV45. Quantitative differences between all three datasets suggest that the target antigen and its context impact on apparent vaccine antibody specificity.

**Table 2:** Purified memory B-cell derived IgG neutralize L1L2 PSV and bind L1L2 PSV and L1 VLP.

			Concentration of purified IgG (μg/mL)^a^											
			PSV Neutralization				PSV Binding				VLP Binding			
Vaccine	ID	Purified IgG (μg/mL)	PSV16	PSV18	PSV31	PSV45	PSV16	PSV18	PSV 31	PSV45	VLP16	VLP18	VLP31	VLP 45
Cervarix	5	74.3	0.02	0.05	1.38	4.03	0.19	2.39	8.51	-	0.15	0.20	1.54	3.23
	17	58.4	0.03	0.06	1.34	-	0.14	0.65	1.32	-	0.11	0.20	1.01	3.12
	20	51.3	0.01	0.04	6.20	2.32	0.11	0.28	6.00	3.37	0.07	0.09	0.65	0.52
	25	51.2	0.03	0.07	2.30	-	0.32	1.03	6.59	-	0.17	0.50	3.69	10.06
	30	35.6	0.06	0.06	4.04	1.19	0.12	0.17	4.66	3.08	0.08	0.09	3.73	1.89
	34	43.2	0.10	0.06	1.64	5.78	0.50	-	-	-	0.30	0.47	2.38	2.97
Gardasil	7	111.9	0.10	0.40	5.47	-	0.44	1.27	10.17	-	0.25	0.36	1.93	1.41
	17	38.3	0.07	0.59	5.39	-	0.33	1.56	-	-	0.18	0.43	2.00	1.61
	37	69.6	0.03	0.12	3.32	-	0.15	0.96	5.19	-	0.13	0.45	1.76	3.39
	43	39.7	0.11	0.75	-	-	0.63	-	-	-	0.43	-	-	-

^*a*^ Midpoint neutralizing or binding antibody concentration of memory B cell derived IgG (μg/mL) or not achievable (-) with maximum amount of purified IgG tested. PSV, pseudovirus.

## Discussion

This study evaluated three humoral immune measures (neutralizing antibody, binding antibody and memory B cell responses) raised against vaccine (HPV16 and HPV18) and non-vaccine (HPV31 and HPV45) genotypes in order to provide possible insights into the differential protection afforded by the current HPV vaccines, Cervarix^®^ and Gardasil^®^. The study was carried out in the target age group included in national vaccination programmes and would be expected to represent the optimum responses generated by the current generation of HPV vaccines.

As expected, 100% of individuals were seropositive for binding and neutralizing antibodies against the vaccine genotypes following three vaccine doses. The greater magnitude of serum antibody responses against HPV16 compared to HPV18 [12–14, 21, 23, 25, 26], and the increased immunogenicity of the Cervarix^®^ vaccine compared to Gardasil^®^ for these two vaccine genotypes [12, 14], are consistent with published studies. The proportion of individuals positive for detection of vaccine-type (HPV16 and HPV18) specific memory B cells and the magnitude of their responses were higher than those reported in a study of 18–45 year old women examining responses generated by Cervarix^®^ and Gardasil^®^ [12]. This is probably due to the lower age group recruited to this present study, as suggested by memory B cell responses following Gardasil^®^ vaccination of 9–13 year old girls and 16–26 year old women [32]. Vaccine-type specific memory B cell responses tracked well with the magnitude of serum immune measures.

The proportion of responders and magnitude of their responses against non-vaccine HPV31 and HPV45 were higher than those reported from older women [22], allowing a more robust comparison between these immune measures for non-vaccine genotypes. We previously reported that HPV31 and HPV45 neutralizing antibody responses were quantitatively related to their respective vaccine-type response, that Cervarix^®^ responses were higher than those elicited in Gardasil^®^ vaccinees and that the differential cross-protection against non-vaccine genotypes bestowed by the current vaccines [1, 3, 18, 19] was, in part at least, reflected in the differential neutralizing antibody responses against these genotypes [14]. In the present sub-study, all individuals were positive for binding antibodies and >80% of individuals had measurable memory B cell responses against both HPV31 and HPV45, suggesting a robust response to vaccination irrespective of vaccine received. While the magnitude of the binding antibody response was generally in line with the magnitude of the vaccine-type neutralizing antibody response, for each non-vaccine genotype, the magnitude of the memory B cell response was poorly related, particularly for HPV45.

In order to address this discrepancy in a little more detail, we evaluated the purified IgG derived from memory B cells in both antibody neutralization and binding assays. That *in vitro* stimulated memory B cells from vaccinees elicited both L1 VLP binding and neutralizing antibodies of similar specificities to those found in the serum suggests that stimulation of resting memory B cells did not introduce a bias in the derived specificities. We used L1 VLP in this study as they are the immunogens used in the HPV vaccines [2] and the antigen widely used in serological studies of vaccine immunogenicity [13, 22, 25, 26, 32, 33]. However, when we used L1L2 pseudoviruses as the target antigens in antibody binding assays there were differences in the antibody specifiities derived. These data demonstrate that the apparent impact on L1 topography induced by incorporation of the L2 protein, previously shown for murine monoclonal antibodies [38], also applies to those antibodies elicited by the HPV vaccines, particularly for those antibodies with specificity against non-vaccine genotypes.

Taken together these data corroborate observations that the measurement of binding antibodies [25, 26], and possibly enumeration of memory B cells [12], may be useful measures for the evaluation of an HPV vaccine genotype-specific immune response. The detection of neutralizing antibodies against non-vaccine genotypes in the serum and cervicovaginal secretions of vaccinated individuals [14, 20–23], protection against HPV31 pseudovirus transduction in the murine challenge model [39] and a reduced risk of HPV31 infection in vaccinated individuals who generate HPV31 neutralizing antibodies [40] do appear to suggest that such cross-neutralizing antibodies are functionally relevant. However, discrepancies between the assay systems and target antigens suggest that binding antibodies and the enumeration of memory B cells were poorly correlated with the cross-neutralizing antibody response and therefore we consider these measures are unlikely to be useful for evaluating the immune response to non-vaccine genotypes.

A next generation HPV vaccine comprising an extended range of VLP [41] should provide greater coverage than the current bivalent (Cervarix^®^) and quadrivalent (Gardasil^®^) vaccines [42]. The significant cost implications of multivalent vaccines may be mitigated by observations that genotype-specific antibody titers in reduced dosing schedules of the current HPV vaccines are non-inferior to those generated under the standard three dose schedule [43–45]. Although next generation HPV vaccines with increased valency will likely be introduced into national programmes over the coming years these are likely to be prohibitively expensive for many low and middle income countries, at least initially. Furthermore, even in countries that adopt such vaccines in the near future several birth cohorts of girls (and in some cases, boys) have already received the current generation of vaccines. An improved understanding of the immune responses elicited following immunization with the current generation of vaccines, and in particular the relationship between immune measures for both vaccine and non-vaccine genotypes, should improve our ability to track such responses in vaccinated individuals and perhaps anticipate any decline in a protective immune response.

## Supporting Information

S1 Table Supporting Information: Vaccine Measures Minimal Dataset. (XLSX)
